# Evaluation of Bleaching Effect on Tooth Color Change With Different Enamel Thicknesses: An In Vitro Study

**DOI:** 10.1155/ijod/6758360

**Published:** 2026-10-14

**Authors:** Tayebeh Rostamzadeh, Nastaran Sohrabi Vaskeh

**Affiliations:** ^1^ Department of Restorative Dentistry School of Dentistry Guilan University of Medical Sciences Rasht Iran gums.ac.ir; ^2^ School of Dentistry Guilan University of Medical Sciences Rasht Iran gums.ac.ir

**Keywords:** bleaching agents, dental enamel, tooth discoloration

## Abstract

**Aim:**

Tooth bleaching efficiency may be influenced by enamel thickness, but the particular relationship is not well understood. This in vitro study investigated the impact of enamel thickness on color change following bleaching with 40% hydrogen peroxide (HP) or 15% carbamide peroxide (CP).

**Material and Methods:**

Sections were taken from 48 bovine incisors at the cementoenamel junction. Buccal surfaces were cut into 6 × 6 mm^2^ blocks and randomly assigned to four enamel thickness groups: intact, 1, 0.5, and 0 mm (exposed dentin). Each group was separated into HP and CP subgroups. All specimen preparation, thickness adjustments, and bleaching procedures were performed by a single trained operator to ensure consistency and minimize bias. A spectrophotometer (VITA Easyshade V) was used to evaluate color at baseline and after bleaching. ΔE00 and the whitening index (WID) were computed. Statistical analysis was conducted with SPSS v23 (*p*  < 0.05).

**Results:**

Repeated measures indicated no significant ΔE00 change with extra sessions (*p*  > 0.05), although WID considerably improved in all groups (*p*  < 0.05), except for complete enamel (*p* = 0.127). Enamel thickness had a significant effect on findings (*p*  < 0.05). Exposed dentin showed higher ΔE00 (*p*  < 0.05) but lower WID with CP (*p*  < 0.05), while HP was more effective on dentin (*p*  < 0.05). The 0.5 mm groups had limited reaction (lowest ΔE00/WID, *p*  < 0.05). 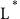 values increased in enamel (*p*  < 0.05), but dropped in dentin. Most groups showed a significant reduction in 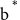 values (*p*  < 0.05). The clinical relevance of WID was supported by the dissociation of ΔE00 (*p*  < 0.05).

**Conclusion:**

Both agents performed best on intact enamel with a thickness of 1 mm. Enamel with a thickness of 0.5 mm had the lowest ΔE00/WID response. Exposed dentin (0 mm) had the highest ΔE00 but required HP for effective whitening. In this in vitro scenario, WID may be a more relevant efficacy measure than ΔE00. This emphasizes the importance of bleaching methods that are thickness‐specific. A single session was sufficient for undamaged enamel; however, several sessions enhanced whitening in thinner enamel and exposed dentin.

## 1. Introduction

A person’s smile is among their most defining esthetic features [1]. Consequently, a growing number of patients seek attractive, perfect smiles, with tooth color being a primary concern [2]. Dental bleaching, a conservative way of whitening stained teeth, is frequently performed with hydrogen peroxide (HP) or carbamide peroxide (CP) at different doses, either in‐office or at home [3].

Tooth color is determined by the combined optical characteristics of both enamel and dentin [4]. Dentin, with its higher chroma, plays a more significant impact on total tooth color, while translucent enamel controls light transmission, influencing the final shade [5]. Although bleaching is a well‐established treatment for tooth discoloration [6], the proportional contribution of enamel and dentin to the total color change remains unclear, despite evidence demonstrating that both tissues are impacted [7–10].

In vital bleaching, the chemical is often administered directly to the enamel surface; hence, many studies focus entirely on the enamel’s whitening response [11]. However, in circumstances of dentin exposure (e.g., due to wear, abrasion, or recession), the bleaching chemical hits dentin directly, requiring examination under both conditions [12]. Existing studies on dentin bleaching are limited and report conflicting results [13]. Although enamel and dentin share similar compositions, differences in their organic and inorganic contents lead to distinct interactions with light [14].

For example, Ma et al. [4] revealed that the absence of enamel improves bleaching efficiency, allowing greater color shift in dentin [15]. Another study verified that bleaching affects both tissues but highlighted the major role of underlying dentin in the ultimate outcome [16]. In contrast, several reports suggest that enamel color has a greater influence on overall tooth color change than dentin [4, 5], while others observed similar whitening effects in both enamel and dentin [7, 10, 17].

Given the scarcity of studies examining how enamel thickness effects bleaching efficacy—and in light of these contradictory findings—the purpose of this study was to explore the effect of bleaching on color change across various enamel thicknesses, including exposed dentin. The null hypothesis was that enamel thickness did not significantly affect ΔE00, whitening index (WID), or color coordinates (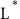, 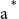, 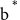) after bleaching with HP or CP. Testing this theory is clinically significant since the effect of enamel thickness on bleaching efficacy is unknown, and understanding this link might help physicians choose optimal bleaching techniques for teeth with different enamel states.

## 2. Materials and Methods

### 2.1. Preparation of Specimens

With the approval of the Ethics Committee of Guilan University of Medical Sciences (IR.GUMS.REC.1403.219), this in vitro study was conducted on 48 bovine incisors. Based on a pilot study [15] with means of 27.31 (intervention) and 28.48 (control), the sample size was estimated using Pocock’s formula with 95% confidence and 90% power, obtaining six participants per group [15]. The effect size was derived from the pilot data; however, no separate a priori power analysis was performed. Bovine incisors were selected due to their similarity in chemical composition and structure to human teeth, as well as their ease of collection and color standardization [18, 19]. After disinfection, the samples were kept in a 0.1% thymol‐buffered solution. The samples were examined under a 4x light microscope (OLYMPUS SZX7, Japan), and any cracks or stains were removed and replaced. For the remainder of the investigation, the specimens were kept in distilled water.

Forty‐eight specimens were randomly allocated to the experimental groups using a computer‐generated randomization list. Stratified randomization was not used because all specimens were obtained from the central buccal surface of bovine incisors with an equal beginning size. All specimen preparation, thickness modifications, and bleaching processes were carried out by a single trained operator to ensure consistency and reduce bias.

### 2.2. Definition of Groups

A double‐faced diamond disk (KG Sorensen Ind. Com Ltd, Cotia, SP, Brazil) mounted on a low‐speed dental handpiece with continuous water irrigation was used to extract the crowns from the roots at the cemento–enamel junction (CEJ) after they had been polished with pumice powder. The mesial, distal, cervical, and incisal thirds of the buccal surface were sliced to produce 6 mm × 6 mm blocks, as seen in Figure 1.

**Figure 1:**
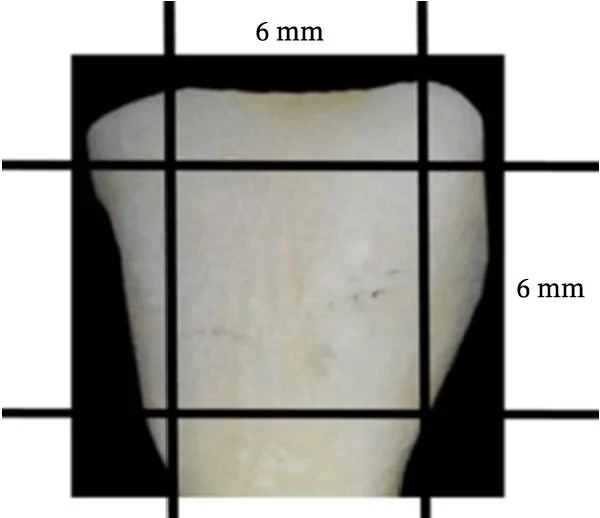
Sectioned dental block.

Using a polishing machine (Arotec Ind. Com., Cotia, SP, Brazil) under continuous water irrigation, the enamel and opposing dentin surfaces were successively abraded with 600‐ and 1200‐grit silicon carbide (SiC) abrasive papers (CarbiMet 2; Buehler, Lake Bluff, IL, USA). Dentin thickness was standardized to 2.0 mm across all specimens as measured with a digital caliper. Enamel thickness was the only factor used to define the experimental groups. In the first group, the enamel surface was preserved at its normal thickness; in the second and third groups, it was abraded to thicknesses of 1 and 0.5 mm, respectively; and in the fourth group, the enamel was completely removed to reveal the underlying dentin (enamel thickness of 0 mm). The enamel thickness was standardized using a digital caliper. Between each abrasive paper application, the specimens were cleaned in an ultrasonic bath (Cleaner ART Ultrasonic) with distilled water.

### 2.3. Bleaching Process

Each group was divided into two subgroups. All specimen preparation, thickness adjustments, and bleaching procedures were performed by a single trained operator to ensure consistency and minimize operator‐related bias. The bleaching gel was applied according to the manufacturer’s instructions as follows: the first subgroup was treated with 40% HP (Opalescence Boost, Ultradent, USA) and the second subgroup with 15% CP (Opalescence PF, Ultradent, USA). The experimental groups are summarized in Table 1, which defines the abbreviations used throughout the manuscript based on enamel thickness (intact, 1, 0.5, and 0 mm) and treatment agent (H: hydrogen peroxide; C: carbamide peroxide).

**Table 1:** Summary of experimental groups.

Abbreviation	Enamel thickness	Dentin thickness (mm)	Treatment agent
EH	Intact (~1.0 mm)	2.0	Hydrogen peroxide
EC	Intact (~1.0 mm)	2.0	Carbamide peroxide
1EH	1.0 mm	2.0	Hydrogen peroxide
1EC	1.0 mm	2.0	Carbamide peroxide
0.5EH	0.5 mm	2.0	Hydrogen peroxide
0.5EC	0.5 mm	2.0	Carbamide peroxide
0EH	0 mm (dentin exposed)	2.0	Hydrogen peroxide
0EC	0 mm (dentin exposed)	2.0	Carbamide peroxide

Three applications of the 40% HP gel were made, separated by 1 week. Twice, for a total of 20 minutes per session, the gel was applied. The 15% CP gel was used for 4 h every day for 2 weeks. Following each application, the gel was removed using cotton‐tipped Johnson & Johnson (São José dos Campos, SP, Brazil) applicators. In order to allow for rehydration, the specimens were properly rinsed under running water after each bleaching procedure, dried with absorbent paper, and kept in artificial saliva (Mucosamin spray, Italy) for a full day prior to color measurements.

### 2.4. Color Measurements

Color readings were performed at baseline (before bleaching) for all groups. Post‐bleaching measurements were then conducted: samples bleached with CP were measured once after the entire 2‐week period, while those bleached with HP were measured after each of the three bleaching sessions and once after the entire process.

Prior to the measurement, the samples were cleaned in an ultrasonic bath with distilled water, laid on a white background, and quantified using a digital spectrophotometer (VITA Easyshade V, Germany). The equipment was calibrated before every use. To assure accuracy, three consecutive readings were taken for each sample.

Color changes (ΔE00) were calculated using the CIEDE2000 formula [20]:

ΔE00=ΔL′KLSL2+ΔC′KCSC2+ΔH′KHSH2+RTΔC′KCSCΔH′KHSH,

where Δ*L*’ represents changes in lightness, Δ*C*’ in chroma, and Δ*H*’ in hue; *S*_*L*_, *S*_*C*_, and *S*_*H*_ are the weighting functions for each component, and *R*_T_ is the interactive term between chroma and hue differences [5].

The CIE 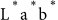
‐based WID, which has overcome the limitations of previous whiteness indexes in validation experiments [6], was also calculated using the following formula [21]:

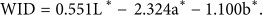




In this system, 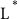 values (ranging from 0 [black] to 100 [white]) indicate lightness; 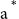 values (−a = greenness, +a = redness) represent the green‐red axis; and 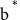 values (−b = blueness, +b = yellowness) represent the blue‐yellow axis. Higher WID values indicate whiter samples, while lower (including negative) values indicate darker samples.

### 2.5. Data Analysis Method

After entering data into SPSS software (version 23), descriptive statistics (mean and standard deviation) were calculated. Data analysis was carried out in accordance with the study goals. Paired *t*‐tests were used to compare within‐group differences (pre‐ and post‐intervention). For studies with more than two repeated color measurements, repeated‐measures analysis of variance (ANOVA) was used. One‐way ANOVA was used to compare groups. Post‐hoc tests (e.g., Tukey’s HSD) were used to uncover particular group differences. All statistical tests were conducted with a significance level of *α* = 0.05.

## 3. Results

Bleaching outcomes varied significantly depending on the enamel thickness and the type of bleaching agent used.

### 3.1. Changes in Color Coordinates (, , )

#### 3.1.1. Lightness ()


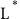
 levels significantly rose in all enamel‐covered groups following HP and CP treatments (*p*  < 0.05, Tables 2 and 3). In contrast, in the exposed dentin groups (0EH and 0EC), 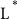 reduced significantly (*p* = 0.015 and *p* = 0.010). This pattern emphasizes the crucial role of enamel in aiding lightness gain during bleaching.

**Table 2:** Means and standard deviation of color coordinates (, , ) of groups treated with HP40%.

Bleaching agent		Hydrogen peroxide 40%			
Time point		Intact enamel (EH)	1 mm enamel (1EH)	0.5 mm enamel (0.5EH)	0 mm enamel (0EH)
L	0	93.41 ± 1.43 ^∗^	93.01 ± 1.77 ^∗^	95.13 ± 2.52 ^∗^	99.33 ± 1.53 ^∗^
	1	99.46 ± 0.68 ^∗^	97.80 ± 1.80 ^∗^	96.58 ± 2.96 ^∗^	88.93 ± 3.60 ^∗^
	2	100.00 ± 0.00 ^∗^	99.43 ± 0.79 ^∗^	98.81 ± 1.24 ^∗^	91.68 ± 4.52 ^∗^
	3	100.00 ± 0.00 ^∗^	99.76 ± 0.57 ^∗^	99.38 ± 0.89 ^∗^	92.46 ± 4.25 ^∗^
a	0	0.76 ± 0.30	1.01 ± 0.14	1.40 ± 0.49	1.36 ± 0.47
	1	0.93 ± 0.64	1.40 ± 0.46	2.03 ± 0.48	1.88 ± 0.37
	2	1.13 ± 0.56	1.15 ± 0.41	1.61 ± 0.14	0.86 ± 0.79
	3	1.46 ± 1.00	1.46 ± 1.00	1.73 ± 0.28	0.66 ± 0.43
b	0	21.58 ± 3.22 ^∗^	28.03 ± 1.70 ^∗^	31.60 ± 4.95 ^∗^	31.70 ± 4.31 ^∗^
	1	26.70 ± 4.79 ^∗^	34.41 ± 2.13 ^∗^	35.35 ± 3.85 ^∗^	28.73 ± 5.00 ^∗^
	2	23.11 ± 4.02 ^∗^	30.00 ± 2.96 ^∗^	31.13 ± 4.50 ^∗^	21.06 ± 4.55 ^∗^
	3	20.96 ± 4.22 ^∗^	20.96 ± 4.22 ^∗^	29.61 ± 2.86 ^∗^	17.85 ± 3.60 ^∗^

*Note:* Asterisks ( ^∗^) denote significant within‐group changes over time (*p*  < 0.05; repeated‐measures ANOVA).

Abbreviation: SD, standard deviation.

**Table 3:** Means and standard deviation of color coordinates (, , ) of groups treated with CP15%.

Bleaching agent		Carbamide peroxide 15%			
Time point		Intact enamel (EC)	1 mm enamel (1EC)	0.5 mm enamel (0.5EC)	0 mm enamel (0EC)
L	0	92.21 ± 1.58 ^∗^	92.83 ± 1.76 ^∗^	92.46 ± 4.14 ^∗^	95.20 ± 7.61 ^∗^
	1	97.70 ± 2.13 ^∗^	99.15 ± 0.87 ^∗^	95.68 ± 3.74 ^∗^	84.96 ± 5.84 ^∗^
a	0	0.60 ± 0.23 ^∗^	1.06 ± 0.47	1.86 ± 1.08	2.25 ± 1.77
	1	1.26 ± 0.41 ^∗^	1.60 ± 0.23	2.16 ± 0.65	1.00 ± 0.73
b	0	24.56 ± 3.94	26.50 ± 1.75	30.66 ± 6.60	34.83 ± 6.09 ^∗^
	1	24.41 ± 4.43	25.63 ± 4.55	25.81 ± 6.40	22.55 ± 5.70 ^∗^

*Note:* 0 = before bleaching; 1 = after bleaching. Asterisks ( ^∗^) denote significant within‐group changes over time (*p*  < 0.05; paired *t*‐test).

Abbreviation: SD, standard deviation.

#### 3.1.2. Red‐Green Coordinate ()

No significant changes in 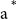 were observed in any HP‐treated group (*p*  > 0.05, Table 1). Among CP groups, only EC showed a significant increase (*p* = 0.006, Table 3); all other CP groups remained unchanged.

#### 3.1.3. Yellowness ()

HP significantly reduced 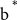 in all groups (*p*  < 0.05, Table 1). In contrast, CP resulted in a significant decline only in the 0EC group (*p* ~ 0.001, Table 3); other CP groups showed downward trends that were not statistically significant.

### 3.2. Color Change (ΔE00)

All groups exceeded the clinically detectable threshold (ΔE00 > 1.8). A one‐way ANOVA with Tukey’s HSD test found significant intergroup variations in ΔE00 values (*p*  < 0.05, Table 4). The exposed dentin groups (0EH and 0EC) consistently had the greatest ΔE00 values, whereas the 0.5 mm enamel groups had the lowest values (Table 4).

**Table 4:** Means and standard deviation of color change (ΔE00) of study groups.

Bleaching agent	Enamel thickness	ΔE1	ΔE2	ΔE3
HP40%	Intact (EH)	6.93 ± 0.93^a^	6.55 ± 1.19	6.54 ± 1.12
	1 mm (1EH)	6.55 ± 1.35^a^	6.70 ± 1.33	7.08 ± 1.31
	0,5mm (0.5EH)	2.34 ± 0.92^b^	2.99 ± 1.34	4.27 ± 1.36
	0 mm (0EH)	8.95 ± 1.71^e^	9.12 ± 1.74	8.94 ± 1.51
CP15%	Intact (EC)	4.64 ± 0.70^f^	—	—
	1 mm (1EC)	6.22 ± 2.10^a^	—	—
	0,5mm (0.5EC)	3.03 ± 1.40^c^	—	—
	0 mm (0EC)	12.74 ± 2.84^d^	—	—

*Note:* Different superscript letters in each column indicate significant between‐group differences (*p*  < 0.05, one‐way ANOVA with Tukey’s HSD). Asterisks ( ^∗^) denote significant within‐group changes over time (*p*  < 0.05; repeated‐measures ANOVA for HP groups).

Abbreviation: SD, standard deviation.

#### 3.2.1. Effects of Further Sessions

Repeated‐measures ANOVA indicated that further bleaching sessions did not substantially affect ΔE00 in any HP‐treated group (*p*  > 0.05 for all), indicating that maximum color alteration was attained after the initial session.

### 3.3. WID

All observed WID changes exceeded the clinical perceptibility (0.85) and acceptability (1.37) thresholds [21].

#### 3.3.1. In HP Groups

Additional bleaching sessions significantly increased WID in groups 1EH (*p* = 0.005), 0.5EH (*p* = 0.010), and 0EH (*p* = 0.001), according to repeated‐measures ANOVA analysis. Only the intact enamel group (EH) showed no significant change across sessions (*p* = 0.127), implying that intact enamel achieves maximum whitening after a single session.

#### 3.3.2. In CP Groups

Paired *t*‐test comparisons between pre‐ and post‐treatment WID values showed a significant increase only in the 0EC group (*p* = 0.026, Table 5). The 0.5EC group exhibited a borderline trend (*p* = 0.057), while the EC and 1EC groups did not show significant changes.

**Table 5:** Means and standard deviation of whiteness index in dentistry (WID) of study groups.

Bleaching agent	Enamel thickness	WID	WID1	WID2	WID3
HP40%	Intact (EH)	23.27 ± 6.17^a^	25.95 ± 4.17	27.04 ± 3.53	28.63 ± 2.76
	1 mm (1EH)	12.78 ± 3.82 ^∗^^a^	18.05 ± 2.30 ^∗^	19.11 ± 4.20 ^∗^	21.81 ± 2.97 ^∗^
	0.5 mm (0.5EH)	9.61 ± 3.93 ^∗^^b^	14.41 ± 5.29 ^∗^	16.45 ± 4.40 ^∗^	18.16 ± 2.52 ^∗^
	0 mm (0EH)	13.02 ± 3.79 ^∗^^a^	16.69 ± 4.70 ^∗^	25.33 ± 4.54 ^∗^	29.76 ± 2.96 ^∗^
CP15%	Intact (EC)	22.39 ± 4.85^a^	24.03 ± 6.20	—	—
	1 mm (1EC)	19.55 ± 2.42^a^	22.72 ± 5.29	—	—
	0,5mm (0.5EC)	12.88 ± 9.55 ^∗^^b^	19.29 ± 7.34 ^∗^	—	—
	0 mm (0EC)	8.91 ± 10.76 ^∗^^b^	19.69 ± 7.35 ^∗^	—	—

*Note:* Different superscript letters in each column indicate significant between‐group differences (*p*  < 0.05, one‐way ANOVA with Tukey’s HSD). Asterisks ( ^∗^) denote significant within‐group changes over time (*p*  < 0.05; repeated‐measures ANOVA for HP groups and paired *t*‐test for CP groups).

Abbreviation: SD, standard deviation.

#### 3.3.3. Intergroup Comparisons

A one‐way ANOVA with post‐hoc Tukey’s HSD test indicated significant variations in WID values among the experimental groups (*p*  < 0.05, Table 5). The intact enamel and 1 mm groups with both bleaching methods produced the highest WID values, whereas the 0.5 mm enamel groups consistently had the lowest WID values. In dentin, HP produced considerably more WID than CP, as evidenced by the comparison of the 0EH and 0EC groups (Table 5).

## 4. Discussion

This study looked at the effect of enamel thickness on tooth color change after bleaching with 40% HP or 15% CP. Four enamel thicknesses were evaluated: entire, 1, 0.5, and 0 mm (exposed dentin). The results showed that enamel thickness had a substantial influence on bleaching efficacy, resulting in the rejection of the null hypothesis.

All groups exhibited a color change (ΔE00) exceeding the clinically acceptable threshold of ΔE00 > 1.8 [22]. Notably, the repeated measures analysis revealed a crucial distinction in how ΔE00 and WID respond to multiple bleaching sessions in HP‐treated groups. While ΔE00 values did not show significant changes with additional sessions across all HP groups, WID values demonstrated a different pattern: only the intact enamel group (EH) failed to show significant improvement in WID with multiple sessions, whereas all other HP‐treated groups (1EH, 0.5EH, and 0EH) exhibited statistically significant increases in WID with successive applications.

The difference between ΔE00 and WID responses reflects their measurement of distinct processes. The consistency of ΔE00 over sessions indicates that the color shift is most successful early in the treatment process, with subsequent sessions yielding diminishing benefits in terms of detectable color difference. This finding is consistent with previous studies indicating that a single session of bleaching is sufficient to achieve the desired ΔE00 [23] and that additional sessions do not result in a substantial further change [24]. Conversely, the continued improvement in WID values in most groups indicates that multiple sessions progressively enhance the whitening efficacy even after maximal color change has been achieved.

The exposed dentin groups (0EC and 0EH) demonstrated the most substantial ΔE00 values in the study (0EC > 0EH), confirming that dentin undergoes profound color changes during bleaching due to its high organic content and permeability [16, 25, 26]. Similarly, based on former investigations, it was expected that bleaching would have a greater effect on dentin alone [5, 10]. However, the whitening outcomes (WID values) revealed an important distinction: although HP caused considerable whitening in exposed dentin (0EH ranking among the highest WID values), CP demonstrated statistically significant decreased efficacy in the 0EC group. This implies that although both treatments elicit significant color change in dentin, HP achieves greater whitening outcomes on exposed dentin surfaces, most likely due to its larger concentration and more violent oxidation of organic pigments in the dentinal matrix [27]. Clinically, however, bleaching agents should not be applied directly to vital exposed dentin due to risks such as reduced fatigue resistance, increased fracture susceptibility, and tooth sensitivity [28, 29]—except in the case of internal bleaching and non‐vital teeth [30].

In the presence of enamel, the higher performance of the EH and 1EH groups in both ΔE00 and WID rankings implies that these enamel thicknesses provide the best clinical setting, permitting significant color change while preserving outstanding whitening efficiency. This exceptional performance can be ascribed to various factors: (1) Sufficient enamel thickness allows for regulated peroxide penetration while retaining structural integrity. (2) The preserved enamel layer has ideal light scattering qualities that improve whitening perception, and (3) the enamel matrix provides enough peroxide exposure to the underlying dentin, avoiding the structural risks associated with exposed dentin. These groups benefit from enamel’s dual role as a semipermeable barrier for peroxide diffusion and an effective light‐diffusing medium that enhances perceived whiteness [10]. A previous study also verified that the thickness of the enamel had a clear impact on the effectiveness of bleaching and that HP penetration was improved after tooth preparation, with a higher ΔE00 mean value than bleaching before preparation [31].

The 0.5 mm enamel groups presented the most challenging scenario, consistently demonstrating the lowest values in both ΔE00 and WID hierarchies. One possible explanation is that this intermediate thickness creates a suboptimal environment for both light scattering and peroxide penetration: it may be too thin to effectively scatter light and enhance perceived whiteness but thick enough to limit peroxide diffusion to the underlying dentin. However, because no direct optical or permeability measurements were taken to prove this mechanism, this interpretation should be regarded as a hypothesis rather than a definitive conclusion unless directly supported by the data. Future research integrating direct optical assessments, permeability tests, and a broader range of enamel thickness levels is needed to support this idea. In contrast to our study, Publio et al. [15] showed that aprismatic enamel had the least color change, indicating the balance between enamel thickness and its permeability.

The examination of color coordinates reveals distinctive patterns: 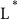 values significantly rose in all enamel‐thickness groups bleached with both HP and CP but reduced in exposed dentin, showing substrate‐dependent lightening effects, correlating with the findings of a prior investigation [10]. This divergence underscores the critical role of enamel as a protective and optical modulator during bleaching. Considering Δ*L*, the groups had a very similar behavior to Δ*E*, representing that 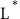 plays a more important role in color change due to bleaching, which is in line with former investigations [31, 32]. The 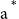 coordinate remained stable across most groups, confirming the selective targeting of yellow‐blue chromophores. Significant 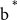 reduction occurred in most groups, except in EC, 1EC, and 0.5EC, where CP’s lower efficacy and enamel masking attenuated the outcome. These results demonstrate the critical interplay between agent type, enamel presence, and optical outcomes. The great changes in 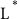 and 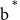 coordinates and minimum changes in a coordinate were also resulted from Caneppele et al. [33] study.

The dissociation between ΔE00 and WID in exposed dentin groups—particularly the poor whitening performance (low WID) despite the substantial color change (high ΔE00) in the 0EC group—underscores that overall color change does not necessarily correlate with perceived whitening. This suggests that WID may be a more clinically relevant outcome measure for bleaching efficacy within the context of this in vitro study rather than conclusively surpassing ΔE00. According to Pérez et al. [21], the whiteness difference thresholds for perceptibility and acceptability are 0.85 WID units and 1.37 WID units, respectively, and the WID changes reported in our investigation exceeded these criteria in the majority of groups. The baseline color can significantly affect the degree of whitening: the whiter the tooth is, the less whitening is expected, and conversely, darker or more yellowish teeth tend to exhibit a greater whitening effect [34].

These findings give critical clinical guidance: (1) HP should be chosen over CP for exposed dentin surfaces to improve whitening outcomes; (2) intact and 1 mm enamel thicknesses produce the best overall results; and (3) teeth with 0.5 mm enamel may necessitate other techniques. These considerations are particularly relevant in clinical scenarios such as cervical abrasion, non‐carious cervical lesions [15], and bleaching prior to resin veneer placement [21], where enamel thickness may be reduced or dentin is exposed. Clinically, however, high‐concentration HP on vitally exposed dentin should be avoided due to safety concern [28, 29]. Future studies should incorporate intraoral simulations, evaluate long‐term color stability, and investigate changes in mineral composition and tissue contribution to color perception.

## 5. Limitations

This study has several limitations that should be considered when interpreting the findings. First, bovine teeth were used instead of human teeth; despite similar composition and structural characteristics [19], differences in permeability, optical properties, and tubular density may limit clinical generalizability. Second, the in vitro design did not replicate the complex oral environment, including salivary flow, temperature changes, pulpal pressure, and mechanical loading, which may affect bleaching outcomes in vivo [32]. Therefore, clinical extrapolation should be made with caution, and future studies incorporating simulated intraoral conditions are warranted.

## 6. Conclusion

Within the limitations of this in vitro study, the following conclusions can be drawn:

1. A single bleaching session sufficed for intact enamel, while both 1 and 0.5 mm enamel layers required multiple sessions to achieve significant whitening improvement.
2. Bleaching efficacy varied significantly across enamel thicknesses (*p* < 0.05).
3. Intact enamel and 1 mm thickness showed optimal whitening (WID) with both agents.
4. 0.5 mm enamel exhibited minimal response (lowest ΔE00/WID).
5. Exposed dentin (0 mm) achieved the highest ΔE00 but required HP for effective whitening.
6. WID may be considered a more clinically relevant efficacy measure than ΔE00 in the context of this in vitro model as all observed WID changes exceeded the established perceptibility and acceptability thresholds.
7. A significant reduction in yellowness (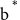) was observed across all groups, while lightness (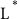) increased in enamel‐covered specimens but decreased in exposed dentin.
8. Given the in vitro design and the bovine model, clinical extrapolation should be approached with caution. While HP is preferable on exposed dentin, it should not be applied directly to vital teeth with exposed dentin due to the possibility of decreased dentin fatigue resistance and sensitivity [28, 29]. Clinical validation is required.

## Author Contributions

**Tayebeh Rostamzadeh:** conceptualization, methodology, supervision, writing – review and editing, project administration. **Nastaran Sohrabi Vaskeh:** investigation, data curation, formal analysis, writing – original draft, visualization.

## Funding

This study did not receive any specific funding.

## Disclosure

All authors have read and approved the final version of the manuscript. Tayebeh Rostamzadeh (Corresponding Author/Manuscript Guarantor) had full access to all of the data in this study and takes complete responsibility for the integrity of the data and the accuracy of the data analysis.

## Conflicts of Interest

The authors declare no conflicts of interest.

## Data Availability

The authors confirm that the data supporting the findings of this study are available within the article.
